# Monomethyl Branched-Chain Fatty Acids Play an Essential Role in Caenorhabditis elegans Development

**DOI:** 10.1371/journal.pbio.0020257

**Published:** 2004-08-31

**Authors:** Marina Kniazeva, Quinn T Crawford, Matt Seiber, Cun-Yu Wang, Min Han

**Affiliations:** **1**Howard Hughes Medical Institute and Department of Molecular, Cellularand Developmental Biology, University of Colorado at Boulder, Boulder, Colorado, United States of America; **2**Laboratory of Molecular Signaling and Apoptosis, Department of Biological and Materials SciencesUniversity of Michigan School of Dentistry, Ann Arbor, MichiganUnited States of America

## Abstract

Monomethyl branched-chain fatty acids (mmBCFAs) are commonly found in many organisms from bacteria to mammals. In humans, they have been detected in skin, brain, blood, and cancer cells. Despite a broad distribution, mmBCFAs remain exotic in eukaryotes, where their origin and physiological roles are not understood. Here we report our study of the function and regulation of mmBCFAs in *Caenorhabditis elegans,* combining genetics, gas chromatography, and DNA microarray analysis. We show that C. elegans synthesizes mmBCFAs de novo and utilizes the long-chain fatty acid elongation enzymes ELO-5 and ELO-6 to produce two mmBCFAs, C15ISO and C17ISO. These mmBCFAs are essential for C. elegans growth and development, as suppression of their biosynthesis results in a growth arrest at the first larval stage. The arrest is reversible and can be overcome by feeding the arrested animals with mmBCFA supplements. We show not only that the levels of C15ISO and C17ISO affect the expression of several genes, but also that the activities of some of these genes affect biosynthesis of mmBCFAs, suggesting a potential feedback regulation. One of the genes, *lpd-1,* encodes a homolog of a mammalian sterol regulatory element-binding protein (SREBP 1c). We present results suggesting that *elo-5* and *elo-6* may be transcriptional targets of LPD-1. This study exposes unexpected and crucial physiological functions of C15ISO and C17ISO in C. elegans and suggests a potentially important role for mmBCFAs in other eukaryotes.

## Abstract 2

Monomethyl branched chain fatty acids (mmBCFAs) are found in bacteria and up through mammals. *C. elegans* produces two mmBCFAs that are essential for growth, suggesting an important role in other eukaryotes

## Introduction

Fatty acids (FAs) belong to a physiologically important class of molecules involved in energy storage, membrane structure, and various signaling pathways. Different FAs have different physical properties that determine their unique functions. Among the most abundant in animal cells as well as the most studied are those of long-chain even-numbered saturated and unsaturated FAs.

**Figure 1:**
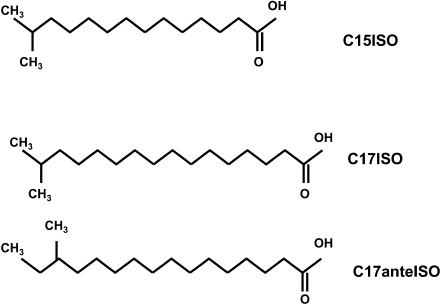
Structure of mmBCFAs of 15 and 17 Carbons C15ISO, 13-methyl myristic acid; C17ISO, 15-methyl hexadecanoic acid; C17anteISO, 14-methyl hexadecanoic acid. Other mmBCFAs mentioned in the text are the following: C13ISO, 11-methyl lauric acid; C15anteISO, 12-methyl tetradecanoic acid. C15ISO and C17ISO are readily detectable in *C. elegans.*

C15ISO and C17ISO are saturated tetradecanoic and hexadecanoic FAs with a single methyl group appended on the carbon next to the terminal carbon (Figure 1). Monomethyl branched-chain FAs (mmBCFAs) in ISO configuration as well as in anteISO configuration (methyl group appended on the second to the terminal carbon) also seem to be ubiquitous in nature. They are present in particularly large quantities in various bacterial genera, including cold-tolerating and thermophilic species (Merkel and Perry 1977; Annous et al. 1997; Ferreira et al. 1997; Batrakov et al. 2000; Jahnke et al. 2001; Groth et al. 2002; Nichols et al. 2002). There, mmBCFAs contribute to the membrane function, regulating fluidity (Rilfors et al. 1978; Suutari and Laakso 1994; Cropp et al. 2000; Jones et al. 2002) and proton permeability (van de Vossenberg et al. 1999).

Although comprehensive reports on mmBCFAs in eukaryotes are lacking, sporadic data indicate that they are present in the fungi, plant, and animal kingdoms (Garton 1985; Seyama et al. 1996; Martinez et al. 1997; Cropp et al. 2000; Wolff et al. 2001; Destaillats et al. 2002). In mammals, mmBCFAs have been detected in several tissues, including skin (Aungst 1989), Vernix caseosa (Nicolaides and Apon 1976), harderian and sebaceous glands (Nordstrom et al. 1986), hair (Jones and Rivett 1997), brain (Ramsey et al. 1977), blood (Holman et al. 1995), and cancer cells (Hradec and Dufek 1994). The fact that mmBCFAs are present in a wide variety of organisms implies a conservation of the related metabolic enzymes and consequently important and perhaps unique functions for these molecules (Jones and Rivett 1997). Nevertheless, their physiological roles and metabolic regulations have not been systematically studied and thus remain fragmentary. It was found that C21anteISO is the major covalently bound FA in mammalian hair fibers. A removal of this FA from its protein counterparts results in a loss of hydrophobicity (Jones and Rivett 1997). Other studies indicated that C17anteISO esterified to cholesterol binds to and activates enzymes of protein biosynthesis (Tuhackova and Hradec 1985; Hradec and Dufek 1994). A potential significance of mmBCFAs for human health is associated with a long-observed correlation between amounts of these FAs and disease conditions such as brain deficiency (Ramsey et al. 1977) and cancer (Hradec and Dufek 1994). More recent studies have revealed a role of another mmBCFA, C15ISO, as a growth inhibitor of human cancer where it selectively induces apoptosis (Yang et al. 2000). Given how important these FA molecules may be and how little is known about their biosynthesis and functions in eukaryotes, it is an opportune problem to study.

De novo synthesis of long-chain mmBCFAs described for bacteria is quite different from the biosynthesis of straight-chain FAs (Smith and Kaneda 1980; Oku and Kaneda 1988; Toal et al. 1995). While the latter uses acetyl-coenzyme A (acetyl-CoA) as a primer condensing with a malonyl-CoA extender, branched-chain FA synthesis starts with the branched-chain CoA primers derived from the branched-chain amino acids leucine, isoleucine, and valine. To synthesize branched-chain FAs, organisms must have a system for supplying branched-chain primers along with the enzymes utilizing them (Smith and Kaneda 1980). No such enzymes have been previously characterized in vivo for any eukaryotic organisms.

Here we describe our approach to characterize the biosynthesis and function of mmBCFAs using the free-living nematode *Caenorhabditis elegans.* Combining genetic, molecular, and biochemical analyses, we show that the worm is not only able to synthesize mmBCFAs de novo but is also absolutely dependent on these FA species for its growth and development.

## Results/Discussion

### C. elegans Synthesizes Branched-Chain FAs De Novo and Uses Two FA Elongation Enzymes to Produce C15ISO and C17ISO

In characterizing FA elongation in *C. elegans,* we identified eight sequences homologous to the yeast long-chain FA elongation enzymes (Kniazeva et al. 2003). To test for their possible functions in vivo, we applied RNAi to the corresponding genes, followed by an analysis of FA composition in whole animals using gas chromatography (GC). RNAi treatment of four genes—*elo-3* (D2024.3), *elo-4* (C40H1.4), *elo-7* (F56H11.3), and *elo-8* (Y47D3A.30)—did not produce any notable phenotypes, whereas suppression of *elo-1* (F56H11.4) and *elo-2* (F11E6.5) affected the elongation of straight long-chain saturated and polyunsaturated FAs (Beaudoin et al. 2000; Kniazeva et al. 2003).

**Figure 2:**
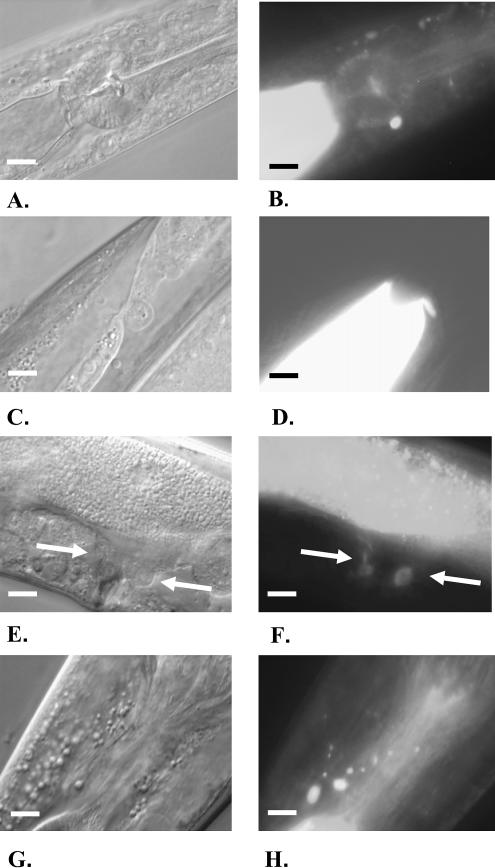
The Expression of *elo-5Prom*::*GFP* and *elo-6Prom*::*GFP* Constructs in Wild-Type Worms (A, C, E, and G) DIC images; (B, D, F and H) fluorescence images. (A–D) Strong expression of the *elo-5Prom*::*GFP* construct in the gut and in the head is shown. (E–H) The expression of the *elo-6Prom*::*GFP* construct in the gut, vulvae (white arrows), and nerve ring is shown. Scale bars, 100 μm.

Surprisingly, the RNAi treatment of the two remaining genes, *elo-5* (F41H10.7) and *elo-6* (F41H10.8), affected the levels of branched-chain FA. Transcriptional reporter constructs (*elo-5Prom*::*GFP* and *elo-6Prom*::*GFP*) indicated that both genes are expressed in the gut (Figure 2). In addition, *elo-5* was expressed in unidentified head cells and *elo-6* was expressed in neurons, pharynx, and vulva muscles.

**Figure 3:**
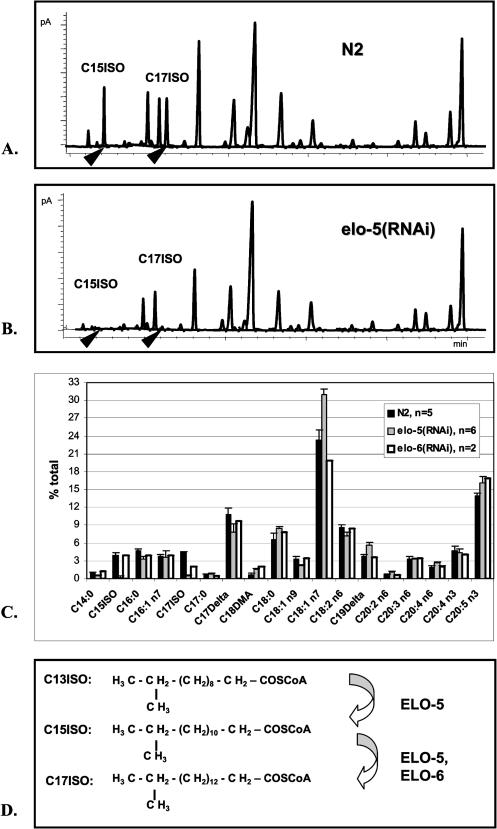
RNAi Treatment of *elo-5* and *elo-6* Significantly Alters the FA Composition (A and B) GC profiles showing the FA composition in the wild-type strain (Bristol N2) containing the RNAi feeding control vector and in the *elo-5(RNAi)* feeding strain. Arrowheads point to the peaks corresponding to C15ISO and C17ISO. (C) Comparison of FA composition in three strains: wild type, *elo-5(RNAi),* and *elo-6(RNAi).* C17ISO is decreased in both RNAi strains, while C15ISO is only decreased in *elo-5(RNAi).* (D) Suggested elongation reactions catalyzed by ELO-5 and ELO-6 in C15ISO and C17ISO biosynthesis. FAs are elongated by an addition of two carbon groups at a time. Combined data presented in this figure and in the text suggest that ELO-6 acts at the elongation step from C15 to C17, whereas ELO-5 may be involved in the production of both C15ISO and C17ISO.

The RNAi of *elo-6* significantly reduced the amount of only C17ISO, while the RNAi of *elo-5* dramatically reduced quantities of both C15ISO and C17ISO (Figure 3). These results indicate that ELO-5 might be involved in the biosynthesis of C15ISO and possibly also C17ISO, whereas ELO-6 may function in elongating C15ISO to C17ISO (Figure 3C and 3D). To our best knowledge, these are the first enzymes that have been shown to be involved in long-chain mmBCFA biosynthesis in a nonbacterial in vivo system and the first enzymes of the long-chain FA elongation family related to mmBCFA production.

**Figure 4:**
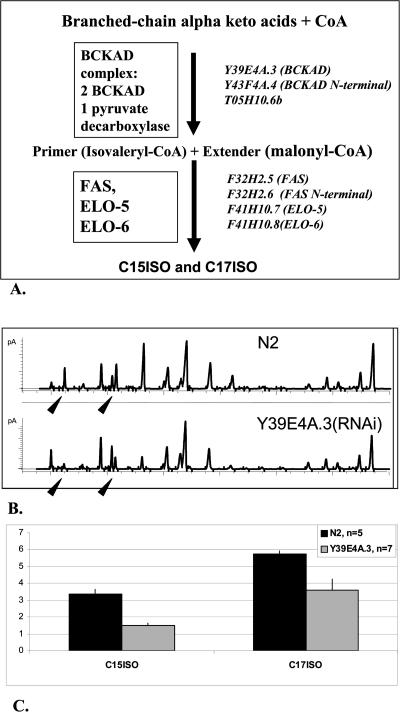
The C. elegans BCKAD Homolog Is Involved in mmBCFA Biosynthesis (A) Early steps of mmBCFA biosynthesis in bacteria, based on Smith and Kaneda (1980), Oku and Kaneda (1988), and Toal et al. (1995). IVD, isovaleryl-CoA dehydrogenase. Predicted corresponding C. elegans genes encoding predicted orthologs were identified (shown in italicized names of reading frames). (B) GC profiles reveal differences in the FA composition in the wild-type animals and animals treated with RNAi of E1 alpha subunit of BCKAD encoded by Y39E4A.3. Black arrowheads point to C15ISO and C17ISO. (C) A summary of several independent preparations shows a significant decrease in both mmBCFAs in the Y39E4A.3 dsRNA-treated animals (*p* = 0.001 and 0.008 for C15ISO and C17ISO, respectively).

In bacteria, mmBCFA biosynthesis utilizes branched-chain α-keto-acids of leucine, isoleucine, and valine to produce mmBCFA acyl-CoA primers that substitute for acetyl-CoAs in conventional FA biosynthesis (Oku and Kaneda 1988). Key enzymes engaged in synthesizing the mmBCFA acyl-CoA primers are branched-chain aminotransferase (BCAT) and the branched-chain α-keto-acid dehydrogenase (BCKAD) complex (Figure 4A). The elongation of the mmBCFA backbone is then carried out by fatty acid synthetase (FAS).

The ability of C. elegans to grow on the chemically defined axenic medium CbMM (Lu and Goetsch 1993), which lacks the potential mmBCFA precursors, has suggested that the animals can synthesize mmBCFA de novo. If so, a disruption of the BCKAD complex could affect mmBCFA levels. We identified a predicted C. elegans protein, Y39E4A.3, with significant sequence homology to the E1 alpha subunit of BCKAD (Y39E4A.3 scores expect value 8e-50 on 57% of the length with the Bacillus subtilis BCKAD and 1.4e-134 on 88.4% of the length with the Homo sapiens BCKADs). RNAi of Y39E4A.3 led to a significant decrease in C15ISO and C17ISO production (Figure 4B and 4C). RNAi suppression of another predicted component of the BCKAD complex, pyruvate dehydrogenase (T05H10.6), resulted in a similar decrease in C15ISO and C17ISO (unpublished data), indicating a role for the C. elegans BCKAD protein in long-chain mmBCFA biosynthesis. Thus, C. elegans appears to use the same initial reactions to produce mmBCFAs as bacterial cells. In addition, the worm uses enzymes of the FA elongation family, ELO-5 and ELO-6, to complete the pathway.

A connection between BCKAD functions and mmBCFA quantities has been previously reported in humans (Jones et al. 1996). Normally hair fibers are densely covered with C21anteISO, which contributes about 38.2% to the total hair FAs (Jones and Rivett 1997). It was observed that patients with maple syrup urine disease, which is caused by an inherited mutation in the BCKAD gene, had a drastically reduced level of mmBCFAs in their hair. Together, these data suggest that long-chain mmBCFA biosynthesis could be similar in bacteria, *C. elegans,* and human.

### Blocking ELO-5 Function Causes Growth and Developmental Defects

**Figure 5:**
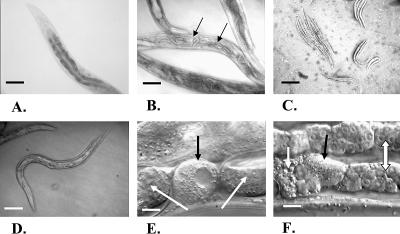
RNAi Treatment of *elo-5* Causes L1 Arrest and Other Physiological Defects (A–C) Nomarski images of worms grown from eggs placed on RNAi plates. Scale bars, 100 μm. (A) Young adults had normal morphology and growth rates. (B) On the second day of adulthood, these animals displayed an egg-laying defect; eggs hatched inside the worms. Arrows point to the late embryos and hatched larvae inside a worm. (C) F1 generation arrested uniformly at the first larval stage (L1), and larvae arrested for 4–5 d died. (D–F) Images of worms derived from late larvae (L2–L4) placed on the RNAi plates. (D) The F1 progeny of worms developed from the treated larvae had smaller size and a scrawny morphology compared to the wild type shown in (A). Scale bar, 100 μm. (E) These animals produced very few oocytes, some of which gave rise to embryos and L1 worms. White arrows indicate embryos. Some oocytes remained unfertilized (black arrow). Scale bar, 10 μm. (F) The proximal part of the gonads undergoes deterioration resulting in sterility. The white arrow indicates spermatica, the black arrow shows an abnormally amorphous oocyte, and the two-way arrow points to the clumsy gonad arm that is finely ordered in wild-type animals. Scale bar, 10 μm.

While the suppression of *elo-6* activity by feeding double-stranded RNA (dsRNA) to wild-type animals did not cause obvious morphological or growth defects, the suppression of *elo-5* resulted in more pronounced phenotypes (Figure 5). Worms originating from wild-type eggs laid on the *elo-5(RNAi)* plates displayed no obvious growth or morphological abnormality until the second day of adulthood, when they developed an egg-laying defect (Figure 5B). Eggs of the next generation hatched on time but the progeny arrested at the first of the four larval stages (L1). The small larvae maintained morphological integrity and could survive on a plate for up to 3–4 d. The arrest was only observed in progeny of parents exposed to *elo-5* RNAi at the L1 stage.

When parental animals were subjected to *elo-5* RNAi at later larval stages (L2–L4), their progeny did not arrest in L1 but continued to develop into adulthood. These animals had no obvious defects in locomotion, pharyngeal pumping, intestinal contractions, chemotaxis response, touch sensitivity, or general anatomy (unpublished data). However, the growing worms became progressively sick (Figure 5D–5F). The gonads appeared normal at the L4 and early adult stages, but after fertilization of one to ten oocytes, oogenesis became impaired. Gonad degeneration began with a pronounced vacuolization in the midsection of the gonad followed by the appearance of disorganized clumps of nuclei in the proximal part. An egg-laying defect became apparent and only a few progeny arose from these worms, which then arrested at L1. The development of the *elo-5* RNAi phenotypes is likely due to a gradual elimination of the ELO-5–associated functions. Our data suggest that these functions are crucial for larval growth and development.

We also obtained a likely null mutant of the *elo-5* gene, *elo-5(gk208),* which has a 245-bp deletion eliminating the predicted first exon (Genome Science Center, BC Cancer Research Center, Vancouver, British Columbia, Canada). This allele phenocopies the L1 arrest phenotype of the *elo-5(RNAi)* animals.

### A Deficiency of C15ISO and C17ISO FAs Is Solely Responsible for the Defects Caused by *elo-5(RNAi)*

We reasoned that if the defects observed in the *elo-5(RNAi)* animals resulted directly from the deficiency of C15ISO and C17ISO, then feeding these worms with C15ISO and C17ISO should mask a shortage of endogenous C15ISO and C17ISO and permit the animals to grow normally. As predicted, the C17ISO and C17anteISO supplements rescued the *elo-5* RNAi defects (in 52 of 60 and 58 of 60 plates, respectively). A partial rescue was observed on the plates supplemented with C15ISO and C15anteISO (23 of 38 and 20 of 28 plates, respectively). Corroborating results were obtained when homozygous *elo-5(gk208)* animals supplied with C17ISO grew normally. In sharp contrast, neither saturated or mono- or polyunsaturated FA molecules (C16:0, C16:1 n7, C17:0, C18:3 n6), mmBCFAs with shorter or longer backbones (C13ISO, C18ISO, C19ISO), nor polymethyl branched phytanic acid were able to rescue or reduce defects (0 of 30 plates in each experiment). Therefore, we have determined that only dietary 17-carbon mmBCFAs are competent to bypass the biochemical defect caused by loss of ELO-5 function.

**Figure 6:**
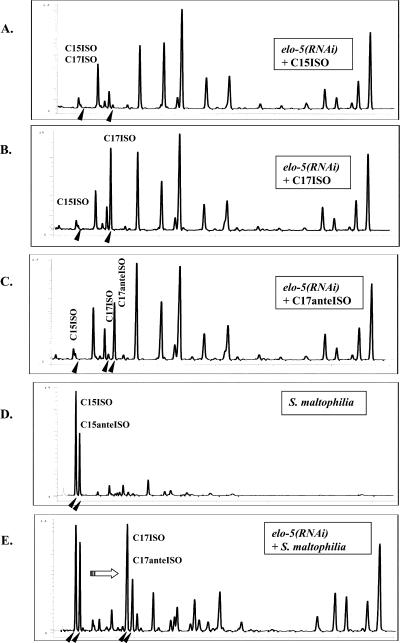
The FA Composition in Worms Maintained on *elo-5* RNAi Plates Supplemented with FA or with S. maltophilia Enriched with C15ISO and C15anteISO FA Black arrowheads indicate positions of mmBCFAs. (A) Animals grown with C15ISO supplements were partially rescued to the wild-type phenotype; however, no accumulation of C15ISO or its elongation to C17ISO was detectable. (B and C) Animals grown with the (B) C17ISO or (C) C17anteISO supplements were fully rescued. Peaks corresponding to C17ISO and C17anteISO are prominent. (D) The FA composition in *S. maltophilia.* Arrowheads point to the major FAs, C15ISO and C15anteISO. (E) The *elo-5(RNAi)* animals are able to elongate dietary C15ISO and C15anteISO into C17ISO and C17anteISO. Arrowheads indicate mmBCFAs. The horizontal arrow illustrates the elongation from C15 to C17 mmBCFA.

GC analysis of FA composition in *elo-5(RNAi)* worms grown on supplemented plates revealed that only C17ISO and C17anteISO are significantly incorporated into lipids (Figure 6A–6C). Because the addition of C15ISO did not result in elongation to C17ISO (Figure 6A), we wanted to determine whether ELO-6 was capable of extending an FA backbone in the absence of ELO-5, or whether the supplied free FA molecules could enter a different metabolic pathway, for instance, a degradation pathway. To distinguish between these two possibilities, we added mmBCFA-producing bacteria on top of the *elo-5(RNAi)* feeding Escherichia coli strain (HT115), which lacks mmBCFAs. This mmBCFA-producing strain was identified by chance; we noticed that in the presence of a certain bacterial contaminant the animals could overcome the *elo-5(RNAi)* effects. Using a rapid bacterial identification method (Lane et al. 1985), we determined the contaminant to be *Stenotrophomonas maltophilia.* GC analysis revealed that this bacterial strain produced a high quantity of C15ISO and C15anteISO but not 17-carbon mmBCFAs (Figure 6D). GC analysis of *elo-5(RNAi)* animals fed with S. maltophilia indicated that they not only accumulated bacterial C15ISO and C15anteISO but also efficiently elongated these FA species to C17ISO and C17anteISO, which are absent in S. maltophilia (Figure 6D and 6E). This suggested that elongation from C15ISO to C17ISO mmBCFA was not impaired in the *elo-5(RNAi)* animals. Therefore, ELO-6 function remains intact in *elo-5(RNAi).* Apparently, C15ISO added to the plates could not be utilized by ELO-6 whereas C15ISO-CoA and/or C15anteISO-CoA originating from the bacterial food could, suggesting that free and esterified mmBCFAs were likely to enter alternative pathways.

The essential roles of C15ISO and C17ISO were also supported through an examination of the *elo-5(gk208)* deletion mutant. The homozygous mutants grew without any obvious morphological defects when maintained on the plates supplemented with C17ISO or seeded with *S. maltophilia.* However, removal of the FA supplements or S. maltophilia by bleaching resulted in the same L1 arrest phenotype seen for the *elo-5(RNAi)* worms.

### L1 Arrest of the *elo-5(RNAi)* Animals Is Reversible and Related to the Variations in Levels of C17ISO during Development

We then asked if *elo-5(RNAi)* animals arrested at the L1 stage could be recovered by adding the 17-carbon mmBCFA supplements. Indeed, C17ISO and C17anteISO could effectively release L1 larvae from the developmental arrest; about 50% of 2-d-arrested and 1% of 4-d-old L1 were rescued to full growth and proliferation. Since C17anteISO could not be detected in the laboratory animals under normal conditions of culturing, C17ISO appeared to be the principal molecule conveying the ELO-5 function. Therefore, the L1 arrest of the C17ISO-depleted worms is both completely penetrant and reversible, indicating that C17ISO plays a critical role in growth and development at the L1 stage.

**Figure 7:**
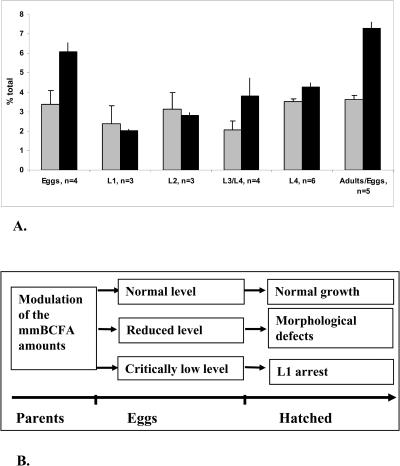
A Fluctuation of the C17ISO Amounts in Development (A) Relative amounts of C15ISO and C17ISO in the worm samples collected at different developmental stages. The amount of the mmBCFA molecule is presented as the percentage of total FA in each sample. Grey bars, C15ISO; black bars, C17ISO. (B) Proposed relationship between the levels of mmBCFA during development and the RNAi effects. Depending on the time of RNAi onset, the amount of C17ISO in F1 eggs varies. If *elo-5* is suppressed in parental animals after they have begun to synthesize mmBCFA, then their eggs will have a reduced C17ISO level that is still above the critical low level, which permits these animals to grow but causes them to display gonadal defects. These worms produce a small number of progeny that is then arrested in L1. If parental animals are treated with *elo-5(RNAi)* right after hatching, they are unable to initiate mmBCFA biosynthesis and the levels of C15ISO and C17ISO in their eggs are reduced to below the critical low level, resulting in L1 arrest of their progeny.

The analysis of the FA levels of staged worms revealed that the C17ISO level increases gradually from a relatively low level at L1 to its peak in gravid adults containing eggs (Figure 7A). Based on the analysis of green fluorescent protein (GFP) reporter constructs (unpublished data) and in situ hybridization data (results from NextDB by Y. Kohara, Tokyo, Japan), neither *elo-5* nor *elo-6* is significantly expressed in eggs or L1. Therefore, C17ISO likely accumulates in embryos during oogenesis. It may be directly transported from gut to gonads, since both ELO-5 and ELO-6 were expressed mainly in the gut and since feeding C17ISO rescued the *elo-5* mutant phenotypes. When RNAi-mediated disruption of *elo-5* occurs at the L1 stage of a parent and consequently blocks C17ISO synthesis from that stage on, the eggs and L1 animals of the next generation are expected to contain a critically low concentration of C17ISO, halting further development. Because the arrested L1 can be rescued by a dietary supply of the mmBCFA, the deficiency is not likely to cause critical defects during the embryonic and early postembryonic periods.

If *elo-5* RNAi is applied to the parent worms at or after the L2 larval stage, when the amount of C17ISO has already been elevated and/or the RNAi effect is less penetrant, the progeny may receive sufficient C17ISO to pass the L1 arrest stage. The resulting animals, however, become visibly unhealthy at the L4 and adult stages as mentioned earlier, suggesting that C17ISO also plays a role in late developmental stages.

Based on these results, we propose a relationship between the amounts of C17ISO and developmental stages (Figure 7B). In this model, the level of C17ISO is monitored at the first larval stage and the decision is made whether to proceed or pause in development. The analysis of GC data from staged animals has also indicated that the variation of the C17ISO level is correlated with only two other FA species, suggesting a potential compensatory and coregulatory mechanism.

### The C17ISO Level Correlates with the Levels of Two Other FAs during Development

**Figure 8:**
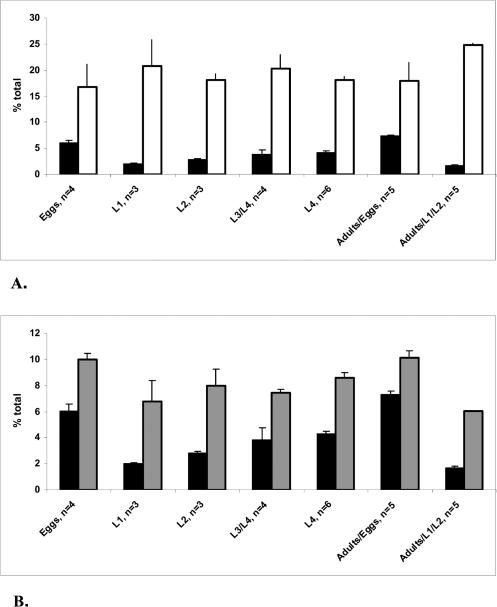
Correlation between the Level of C17ISO and the Levels of Linoleic and Vaccenic Acids during Development Graphical illustrations of the correlation between the levels of C17ISO and (A) vaccenic acid and (B) linoleic acid. Data were obtained by GC analysis of synchronized populations of worms. Combined with the GC measurements generated from 50 additional samples (see Materials and Methods), these data were used to calculate correlation coefficients: CORREL _C17ISO/C18:2 n6_ = 0.82772, T-TEST = 6.54814 × 10^−7^, and CORREL _C17ISO/C18:1 n7_ = −0.85162, T-TEST = 4.74094 × 10^−5^. Black bars, C17ISO; white bars, vaccenic acid; grey bars, linoleic acid.

FA homeostasis implies that relative amounts of various FA species are coordinated and balanced for optimal performance. To obtain information that may help us understand why and how numerous FAs and their specific metabolic enzymes are maintained in nature, we carried out analysis to determine a possible correlation between changes in the levels of C17ISO and other FAs detected in worms. We have analyzed a large amount of GC data (*n* = 50) obtained from mixed populations of wild-type animals where the fractions of eggs, larvae, and adults randomly varied. We also included GC data separately obtained from staged worms: eggs, L1, L2, L3, L4, and gravid adults. We found that the amounts of C17ISO significantly correlated with only two other FA molecules: linoleic acid (C18:2 n6) and vaccenic acid (C18:1 n7) (Figure 8). A potential physiological significance of these correlations is intriguing.

The observed negative correlation between the levels of C17ISO and C18:1 n7 throughout development may indicate a compensatory adjustment important for physiological functions, such as retention of the cell membrane physical properties. mmBCFAs and monounsaturated straight-chain FAs have been previously implicated in regulating membrane fluidity, which depends on the ratio of saturated FA to monounsaturated and branched-chain FA content in bacterial cells (Rilfors et al. 1978; Suutari and Laakso 1994; Cropp et al. 2000). An elevation in monounsaturated FA amounts in response to the decrease of branched-chain FAs, but not vice versa, was observed in Streptomyces avermitilis (Cropp et al. 2000), suggesting that monounsaturated FAs may sense a state of membrane fluidity.

In the *elo-5(RNAi)*-treated worms, a substantial loss of C15ISO and C17ISO is also accompanied by a change in the FA composition, most noticeably by the elevation in C18:1 n7 (see Figure 3C), a result consistent with the above observation. To estimate the effect of the C15ISO and C17ISO deficiency on the membrane saturation, the saturation index (SI = [saturated FA]/[mmBCFA + monounsaturated FA]) was calculated. No significant differences were detected in *elo-5(RNAi)* worm compared to wild type (SI = 0.325 ± 0.011 [*n* = 6] and SI = 0.320 ± 0.032 [*n* = 5], respectively). Therefore, *elo-5(RNAi)* may not cause massive cell membrane dysfunction.

A positive correlation between the amounts of C17ISO and C18:2 n6 may suggest a potential common function during development. In addition to the importance of linoleic acid as a substrate for polyunsaturated FA biosynthesis, its hydroxylated fatty acid derivative (HODEs) is known as a signaling molecule affecting chemotaxis (Kang and Vanderhoek 1998), cell proliferation (Eling and Glasgow 1994), and modulation of several enzymatic pathways (Hsi et al. 2002). A correlation between C17ISO and linoleic acid may also suggest a similar regulation of biosynthesis of the two molecules.

The changes in the FA composition associated with a decrease in C15ISO and C17ISO indicate that the metabolism of straight-chain FA species is responsive to the mmBCFA levels and suggest a cross regulation. Interestingly, in the *elo-5(RNAi)* animals fed with C15ISO or C15anteISO containing bacterial supplement *(S. maltophilia),* the FA composition was significantly altered (see Figure 6E). It appears that mmBCFAs become principal components in a range of 16–18-carbon FAs. This suggests that large quantities of mmBCFAs are not toxic. In contrast, because these worms grow and proliferate well, mmBCFAs seem to be efficient substitutes for saturated and monounsaturated straight-chain FAs.

### The Worm SREBP Homolog Controls Production of Branched-Chain FAs

In mammals, straight-chain FA biosynthesis depends on the 1c isoform of sterol regulatory element binding protein (SREBP-1c), which promotes the expression of FA metabolic enzymes (Edwards et al. 2000; Horton 2002; Matsuzaka et al. 2002). There is only one protein in C. elegans that is homologous to mammalian SREBPs, Y47D3B.7 (the gene has been named *lpd-1,* for “lipid depleted 1”) (McKay et al. 2003). McKay and coauthors have shown that worms treated with *lpd-1* RNAi display a lipid-depleted phenotype. They have also shown that *lpd-1* regulates the expression of several lipogenic enzymes, acetyl-CoA carboxilase (ACC), FAS, and glycerol 3-phosphate acyltransferase (G3PA) (McKay et al. 2003). Thus, similar to its mammalian homolog, *lpd-1* is involved in straight-chain FA biosynthesis.

**Figure 9:**
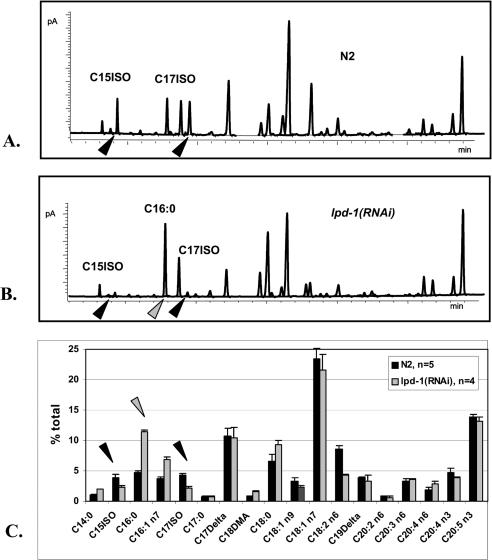
RNAi of the C. elegans SREBP Homolog Alters the FA Composition (A and B) The GC profiles of (A) wild-type and (B) *lpd-1(RNAi)*-treated worms. (C) A summary of several independent GC runs. Bars represent the percentages of total FAs. The levels of C15ISO, C17ISO, and C16:0 are significantly altered by the RNAi treatment. Black arrowheads point to differences in the C15ISO and C17ISO amounts. Grey arrowheads indicate the changes in palmitic acid, C16:0.

We wanted to see if *lpd-1* also plays a role in mmBCFA metabolism. We first applied RNAi to *lpd-1* and determined the FA composition of the mutant worms. As expected, the FA content of treated animals was significantly changed, but surprisingly the most reduced were the levels of C15ISO and C17ISO (Figure 9). Also significantly reduced was the amount of C18:2 n6. In contrast, the C16:0 level was elevated. These data indicate that, in addition to regulating the first steps of global FA biosynthesis through the activation of the ACC and FAS transcription, the worm SREBP homolog regulates mmBCFA elongation as well as desaturation of straight-chain FAs.

As reported previously, disruption of *lpd-1* through a mutation or RNAi injection caused early larval arrest (McKay et al. 2003). The effect of *lpd-1* RNAi feeding in our experiments was apparently less severe. The RNAi-treated animals displayed slow growth, morphological abnormalities, and egg-laying defects but no larval arrest. Supplementing C17ISO to the plates did not significantly rescue these defects.

### LPD-1 and LPD-2 Diverge in Functions

LPD-2 (C48E7.3) is another C. elegans homolog of a mammalian lipogenic transcription factor, CCAAT/enhancer-binding protein (C/EBP). McKay and coauthors have shown that the *lpd-2(RNAi)* and *lpd-1(RNAi)* phenotypes are quite similar; affected worms are defective in growth, pale and scrawny in appearance, and lacking in fat content (McKay et al. 2003). They have also shown that LPD-1 and LPD-2 control the expression of the same lipogenic enzymes: ACC, FAS, ATP-citrate lyase, and G3PA. We tested to see if LPD-1 and LPD-2 function similarly in the regulation of mmBCFA biosynthesis. In contrast to the result from *lpd-1*(RNAi), the FA composition in *lpd-2(RNAi)* worms was not significantly different from that of wild-type animals even though these animals had a noticeably sick appearance (unpublished data). This result suggested that, in addition to having some common targets, LPD-1 and LPD-2 have distinct functions. LPD-1 is important for production of mmBCFAs as well as other very-long-chain FAs, whereas LPD-2 has no specificity for any particular type of FA.

### *elo-5* and *elo-6* Are Likely Targets of LPD-1

The changes in FA composition observed in *lpd-1(RNAi)* would be consistent with downregulation of *elo-5, elo-6* (decrease in mmBCFA), *elo-2* (increase in C16:0) (Kniazeva et al. 2003), and Δ*9-* and/or Δ*12*-desaturase genes (decrease in C18:2 n6). The genes encoding mammalian orthologs of the *C. elegans elo-2* and Δ*9*-desaturase genes are known targets of SREBP-1c (Edwards et al. 2000; Horton 2002; Horton et al. 2002). To examine if *elo-5* and *elo-6* are targets of *lpd-1,* we analyzed the expression of *elo-5, elo-6,* and *lpd-1.*

**Figure 10:**
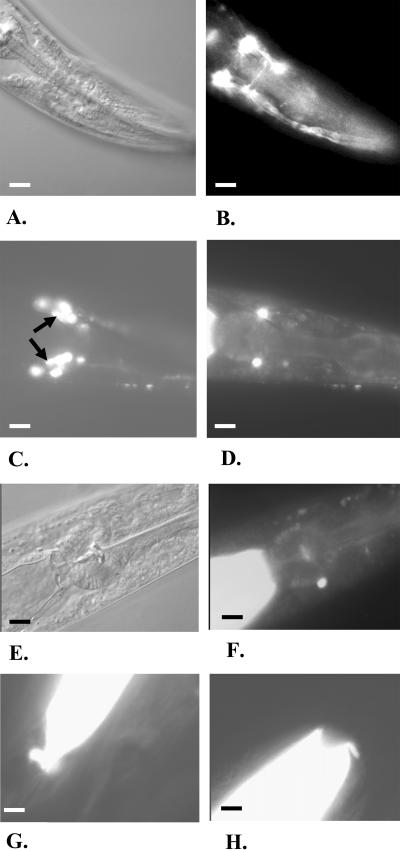
The Expressions of *elo-5* and *lpd-1* Reporter Constructs Are Spatially Similar (A and B) Nomarski and GFP-filtered images of an adult animal containing the *lpd-1Prom*::*GFP* construct, showing strong expression in two symmetrical head neurons, each of which has processes to the nose and around a nerve ring. Scale bars, 10 μm. (C) DiI staining of amphid neurons in *lpd-1Prom*::*GFP* (dsRed filter). Arrows indicate neuronal nuclei shown in (D). Scale bar, 10 μm. (D) GFP expression in the animal shown in (C). Scale bar, 10 μm. (E and F) Nomarski and GFP-filtered images of an animal containing *elo-5Prom*::*GFP,* revealing fluorescence in the similar amphid neuron. Scale bar, 7.5 μm. (G and H) The intestinal and intestinal-muscle GFP expression in (G) *lpd-1Prom*::*GFP* and (H) *elo-5Prom*::*GFP* constructs. Scale bar, 7.5 μm.

Evaluation of the expression from an *lpd-1Prom*::*GFP* fusion construct (a gift of J. Graff) in transgenic animals revealed that, in addition to the previously reported expression in intestinal cells (McKay et al. 2003), the construct is strongly expressed in a subset of head neurons (Figure 10A—Figure 10D). Using a lipophilic dye, DiI, which highlights chemosensory ciliated neurons, we identified these neurons as amphids (Murphy et al. 2003). In the strains carrying *elo-5Prom*::*GFP* and *elo-6Prom*::*GFP* reporter constructs, GFP fluorescence was also detected in the gut and several head neurons, including amphid neurons (Figure 10E–10H and Figure 2).

**Figure 11:**
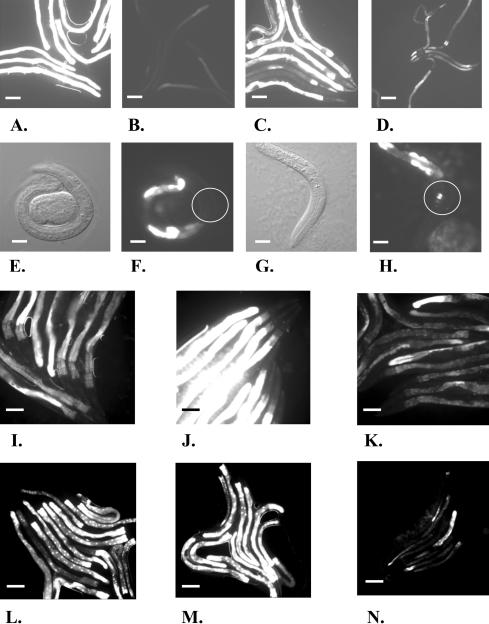
The Expression of GFP Fusion Constructs Suggests the Involvement of *lpd-1, acs-1,* and *pnk-1* in mmBCFA Biosynthesis (A–D) *elo-5Prom*::*GFP* expression is downregulated in the *lpd-1(RNAi)* background. Scale bars, 100 μm. (A and C) GFP-filtered images of (A) *elo-5Prom*::*GFP* and (C) *elo-6Prom*::*GFP* in wild-type animals, showing the characteristic bright intestinal fluorescence. (B and D) GFP-filtered images of (B) *elo-5Prom*::*GFP* and (D) *elo-5Prom*::*GFP* in *lpd-1(RNAi)* animals, revealing diminished fluorescence in the gut. (E–H) *lpd-1* expression is upregulated in neurons of the *elo-5(RNAi)* animals deficient for C15ISO and C17ISO. Scale bars, 15 μm. (E and F) Nomarski and GFP images of wild-type L1 larvae containing *lpd-1Prom*::*GFP.* (G and H) Nomarski and GFP images of *elo-5(RNAi)*-treated animals (L1 arrested) containing *lpd-1Prom*::*GFP,* showing a visibly brighter fluorescence than that seen in (E) and (F). Circles are centered on the pharyngeal back bulb. (I–K) *acs-1Prom*::*GFP* expression is upregulated in the *elo-5(RNAi)* animals deficient for C15ISO and C17ISO. Panels show GFP images of *acs-1Prom*::*GFP* animals grown on the (I) control, (J) *elo-5(RNAi),* and (K) *lpd-1(RNAi)* plates. The fluorescence from *acs-1Prom*::*GFP* in (J) is significantly stronger than that in (I). Scale bars, 100 μm. (L–N) *pnk-1Prom*::*GFP* expression is upregulated by *elo-5(RNAi)* but downregulated by *lpd-1(RNAi).* Panels show GFP images of *pnk-1Prom*::*GFP* animals grown on the (L) control, (M) *elo-5(RNAi),* and (N) *lpd-1(RNAi)* plates. The fluorescence of the fusion construct is stronger in (M) but weaker in (N) than that in the control (L). Scale bars, 100 μm.

If LPD-1 promotes *elo-5* and *elo-6* expression, then RNAi of *lpd-1* should alter GFP intensity in *elo-5Prom*::*GFP* and *elo-6Prom*::*GFP* reporter strains. The level of GFP expression driven by *elo-5* and *elo-6* promoters is high in conventionally cultured animals. In the worms maintained on the *lpd-1(RNAi)* plates, the expression was noticeably weakened, suggesting a downregulation of the promoter activities (Figure 11A–11D). No significant changes in GFP expression were detected in a control strain containing a *kqt-1Prom*::*GFP* construct that also expresses GFP in head neurons and the gut (unpublished data).

To test if the disruption of FAS, a target of LPD-1 (McKay et al. 2003), could contribute to the observed decrease of C15ISO and C17ISO in *lpd-1(RNAi),* we analyzed FA composition in *FAS(RNAi)* strains. There is one predicted FAS gene, F32H2.5, and its shorter homolog, F32H2.6, in the C. elegans genome. The latter can only encode the N-terminal portion of the protein. These genes share extended nucleotide identity, and RNAi of one could thus possibly affect the other. Consistent with a critical role for FAS in the first steps of FA biosynthesis, the RNAi-mediated disruption of F32H2.5 and F32H2.6 resulted in multiple defects and a lethal growth arrest (unpublished data). The FA composition (the content and relative amounts of various FA species) of the affected animals remained, however, unchanged. This suggests that disruption of FAS does not selectively alter FA biosynthesis and that neither FAS protein is specific for mmBCFA. Therefore, downregulation of FAS by loss of *lpd-1* cannot account for the severe deficiency of mmBCFA in *lpd-1(RNAi).*

Thus, we have shown that disruption of *lpd-1* affects C15ISO and C17ISO biosynthesis. The fact that *lpd-1, elo-5,* and *elo-6* are expressed in the same cells concurrently and that the GFP reporter analysis indicated that *elo-5* and *elo-6* transcription is downregulated in the absence of *lpd-1* suggests that *elo-5* and *elo-6* are likely to be the targets of *lpd-1.*

Since ACC and FAS catalyze the first steps in the biosynthesis of straight-chain FAs while ELO-5 and ELO-6 extend mmBCFA molecules, LPD-1 appears to integrate conventional and “unusual” FA biosyntheses. It seems reasonable to predict that in order to differentiate between these metabolic pathways and mediate compensatory or adaptive changes in FA composition, LPD-1 must interact with other factors such as nuclear receptors activated by specific FA ligands. It is thus important to screen for such interactions to better understand FA homeostasis in *C. elegans.*

### A Reciprocal Correlation between *lpd-1* Expression and mmBCFA Levels

Because mammalian SREBP-1c regulates polyunsaturated FA biosynthesis and is feedback inhibited by polyunsaturated FAs (Jump 2002), we asked if *lpd-1* could be regulated by mmBCFAs at the transcriptional level. Our microarray data (discussed below) indicated a 1.68-fold upregulation of *lpd-1* in the *elo-5(RNAi)* animals, while no changes were detected in its levels between samples from wild-type animals at different developmental stages (see Materials and Methods).

To examine the influence of the mmBCFA deficiency on *lpd-1* expression, we grew the *lpd-1Prom*::*GFP*-containing strain on the *elo-5(RNAi)* and control plates to compare GFP fluorescence. No obvious difference in the GFP expression driven by the *lpd-1* promoter in intestinal cells was detected on the *elo-5(RNAi)* plates versus the control plates. A modest change in the transcription level (1.68-fold) could be masked by a variability of the expression between individual animals and even between individual cells (unpublished data). In contrast to the observation for the intestinal cells, a strong induction of GFP was detected in amphid neurons of *lpd-1Prom*::*GFP*;*elo-5(RNAi)* animals (Figure 11E–11H). This suggests that a chronic deficiency of mmBCFA in *elo-5(RNAi)* animals may transcriptionally stimulate LPD-1 production, at least in neuronal cells.

Collectively, our results suggest that the relationship between *lpd-1* and C15ISO/C17ISO is reciprocal; while downregulation of *lpd-1* transcription results in the C17ISO deficiency, the C15ISO and C17ISO deficiency upregulates *lpd-1* transcription at least in a subset of cells. Therefore, the worm SREBP homolog, LPD-1, may play an important role in mmBCFA homeostasis.

### Screening for Additional Genes Involved in mmBCFA Homeostasis

Because C15ISO and C17ISO play critical roles in animal development and growth, we suspected mechanisms might exist to respond to and regulate their levels. Regulation of mmBCFA homeostasis may involve transcription factors, metabolic enzymes, and transport and binding proteins. It is reasonable to suggest that a deficiency of mmBCFA triggers a compensatory alteration in the expression of these genes. It is also possible that a comparative analysis of global gene expressions between wild-type and mmBCFA-deficient animals may reveal these potential changes and the changes underlying developmental and growth functions of mmBCFA.

We used DNA microarray analysis to compare the total gene expression in *elo-5(RNAi)* and wild-type animals. To select candidate genes, we applied restrictive criteria and excluded genes of which the expression was also changed in the *spt-1(RNAi)* strain (Protocol S1 and Table S1). The *spt-1* (C23H3.4) gene encodes a predicted C. elegans homolog of serine-palmitoyl transferase subunit 1. RNAi of *spt-1* strongly affects the FA composition without reducing the C15ISO or C17ISO levels (unpublished data). The F1 generation of *spt-1(RNAi)* animals developed gonadal and egg-laying defects that were similar to the phenotype of F1 animals from parents treated with *elo-5(RNAi)* at a late larval stage (described earlier; see Figure 5B and 5F). We thought that by deselecting genes that have altered expressions in *spt-1(RNAi),* we would be able to eliminate variations in gene expressions unrelated to the mmBCFA deficiency. Such variations might emerge from altered straight-chain FA metabolism and from general sickness. Here, we discuss the analysis of the first set of candidate genes that are differentially expressed in *elo-5(RNAi)* and may relate to the C15ISO and C17ISO homeostasis.

**Figure 12:**
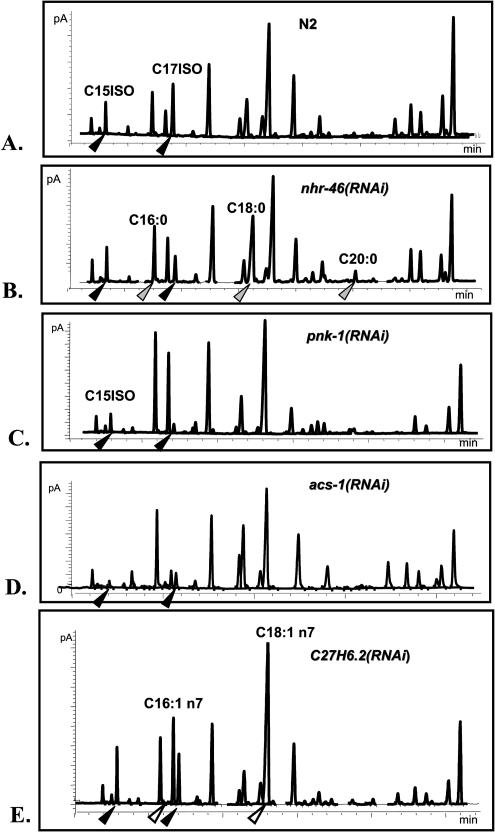
RNAi of Four Candidate Genes with Altered Expression in *elo-5(RNAi)* Worms Affects the FA Composition (A) GC profile of the wild type. (B–E) GC profiles of the RNAi-treated worms. (B–D) RNAi of the three genes resulted in a decrease of the C17ISO or both C15ISO and C17ISO levels, indicated by black arrowheads. In addition, a significant elevation in straight-chain saturated FA levels, indicated by grey arrowheads, is observed in K10C3.6(RNAi). (E) C27H6.2(RNAi) does not cause significant changes in mmBCFA but results in an elevation of straight-chain monounsaturated FA and C18:1 n7, indicated by white arrowheads. Statistical analysis of several GC runs on each of the samples was also carried out (unpublished data).

Twenty-five genes were selected in the screen (Table 1) and each was functionally tested by RNAi and GC analysis for its role in C15ISO and C17ISO metabolism. RNAi of four of these genes (*pnk-1* [C10G11.5], *nhr-49* [K10C3.6], *acs-1* [F46E10.1], and C27H6.2) significantly affected the FA composition (Figure 12). All four genes encoded products structurally homologous to the known proteins (PNK-1, human pantothenate kinase; NHR-49*,* nuclear hormone receptor; ACS-1, very-long-chain FA CoA ligase; and C27H6.2, RuvB-like DNA binding protein).

**Table 1:**
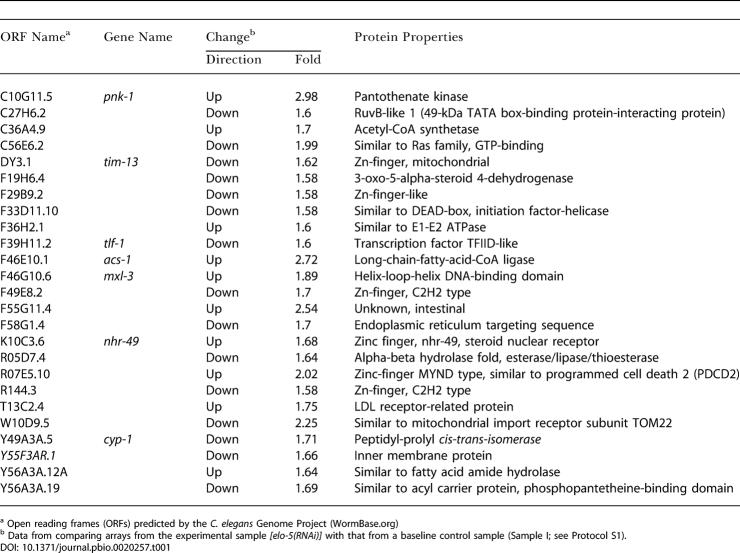
Candidate Genes and Their Encoded Proteins Selected from Microarray Data for Functional Tests (RNAi and GC Analysis)

^a^ Open reading frames (ORFs) predicted by the C. elegans Genome Project (WormBase.org)

^b^ Data from comparing arrays from the experimental sample with that from a baseline control sample (Sample I; see Protocol S1)

### Analysis of the Candidate Genes

Circumstantial evidence suggests that these four candidate genes may be involved in feedback regulation of mmBCFA biosynthesis. First, the expression of these genes is not variable in nature, as judged by a comparison of the microarray data obtained from developmentally different populations of N2 (Protocol S1) as well as for vulval development pathway mutants (data obtained for an unrelated project, J. Chen, personal communication). Second, the direction of the changes for three of the genes is in concordance with the proposed feedback regulation: *pnk-1, nhr-49,* and *acs-1* were upregulated in C17ISO-deficient *elo-5(RNAi)*. Lastly, a functional analysis shows that these three candidate genes are required for the normal level of mmBCFA production (RNAi of the genes affects mmBCFA production). The fourth candidate gene, C27H6.2, affects the level of vaccenic acid (C18:1 n7), which is related to the levels of mmBCFA (see Figure 8), suggesting cross-talk.

To detect potential feedback regulation involving *acs-1* and *pnk-1,* we made reporter strains with GFP expression driven by *acs-1* and *pnk-1* promoters, *acs-1Prom*::*GFP* and *pnk-1Prom*::*GFP,* respectively. These two genes showed a higher degree of upregulation than the other candidates according to the microarray data. In addition, RNAi of these two genes resulted in a significant loss in the mmBCFA fraction. The GFP fluorescence from *acs-1Prom*::*GFP* and *pnk-1Prom*::*GFP* was readily detectable in the gut. Expression of *acs-1Prom*::*GFP* was also detected in the canal-associated neurons in the head neurons and vulval cells. A comparison of synchronized animals grown on the control and *elo-5(RNAi)* plates indicated a significantly brighter fluorescence in the RNAi worms (see Figure 11I, 11J, 11L, and 11M), suggesting upregulation of *acs-1*and *pnk-1* under C15ISO or C17ISO deficiency. These results were in concordance with the microarray data. Moreover, *pnk-1,* but not *acs-1,* seemed to be regulated by LPD-1 because *pnk-1Prom*::*GFP* expression was significantly reduced on *lpd-1(RNAi)* (see Figure 11L and 11N).

It was interesting to note that the *pnk-1* and *acs-1* genes were previously selected in two different screens as potential targets of the *daf-2/daf-16* (Y55D5A.5 and R13H8.1, respectively) pathway. *pnk-1* had been identified in a screen for genes affecting the life span and metabolism of C. elegans through analysis of promoter regions, and it was confirmed as a direct target of DAF-16, a forkhead transcriptional factor (Lee et al. 2003). *acs-1* had been identified in a microarray screen for DAF-16 targets that influence life span (Murphy et al. 2003). A third gene, *nhr-49,* had been previously selected in a screen for fat regulatory genes (Ashrafi et al. 2003). It was shown that RNAi of this gene leads to an increase in fat accumulation in affected animals. Our analysis of *nhr-49(RNAi)* animals showed that reduction of *nhr-49* activity results in upregulation of saturated FA biosynthesis that may contribute to fat accumulation. Although the regulatory path for this process remains unknown, the involvement of *daf-2* has not been ruled out.

A potential link between the candidate genes and DAF-2/insulin signaling is very intriguing. The C. elegans insulin-signaling pathway is involved in sensing nutritional state and metabolic conditions as well as controlling growth and diapause (Kimura et al. 1997; Ailion et al. 1999). As we report in this paper, a mmBCFA deficiency causes transient L1 arrest. This phenotype strikingly resembles L1 arrest of worms hatched in the absence of food (a method commonly used to obtain synchronized animals). An investigation of possible roles for mmBCFA in food sensation and insulin signaling pathways is underway.

Downregulation of the fourth candidate gene, C27H6.2, may result in a significant increase in monounsaturated FA levels (Figure 12). This is consistent with the enlarged fraction of monounsaturated FAs observed in the *elo-5(RNAi)* animals (see Figure 3C). Downregulation of C27H6.2 may have an adaptive effect to compensate for the loss of mmBCFAs in cell membranes. If so, C27H6.2 may be part of a mechanism that senses and tunes physical properties of membranes. C27H6.2 is homologous to RuvB/TIP49a/Pontin52, an evolutionarily conserved protein essential for growth and proliferation (Kanemaki et al. 1997; Bauer et al. 1998; Qiu et al. 1998). Its mammalian ortholog acts as a transcriptional cofactor that binds to β-catenin, TATA-box binding protein, and likely to a number of other diverse transcription factors (Bauer et al. 1998).

### Concluding Remarks

Two mmBCFAs are normally detected in *C. elegans:* C15ISO and C17ISO. A deficiency of these FAs is lethal and cannot be compensated by any other FA present, indicating their crucial importance for growth and development. There are two sources of C15ISO and C17ISO available for worms. First, they possess a system for mmBCFA biosynthesis that includes two FA elongation enzymes, ELO-5 and ELO-6, which are regulated at least in part by the nematode homolog of SREBP-1c *(lpd-1).* Second, worms may obtain mmBCFAs from their diet (bacteria). Therefore, C. elegans is able to produce, activate, transport, and utilize mmBCFAs and is vitally dependent on this system.

The level of C15ISO and C17ISO in eggs appears to be critical for growth and development, as animals depleted of C15ISO or C17ISO completely arrest at the L1 stage. The uniformity and reversibility of the arrest would be consistent with a regulatory role in growth and development for these mmBCFAs or for more complex lipid molecules containing them. However, it cannot be ruled out that the arrest is due to the failure of a metabolic or structural function that is essential for growth and development at the first larval stage. In addition, C15ISO and C17ISO may directly or indirectly regulate genes involved in FA homeostasis. Consistent with this, their deficiency triggers a large alteration in gene expression that may reflect a complex feedback mechanism. Among the potentially responsive genes are transcription factors and metabolic genes.

Ubiquitous and unattended mmBCFAs come forth as physiologically important molecules that regulate essential functions in eukaryotes. Many interesting questions regarding mmBCFAs remain to be addressed. What are the other components of the mmBCFA biosynthetic machinery? What are the components of their transport system? Does an organism have a mechanism by which the mmBCFA level is measured? What are the signaling pathways involved in the mmBCFA responses? How do mmBCFAs exert their physiological function? Do mmBCFAs act alone or as parts of more complex lipids? How are mmBCFAs synthesized in mammals? Lastly, what are the specific physiological functions of mmBCFAs in mammals? Both genetic and biochemical approaches will be taken to address these questions.

## Materials and Methods

### 

#### RNA interference by feeding

The RNAi feeding vectors were either made in our laboratory using Taq PCR and cloning genomic fragments into a double T7 vector, pPD129.36 (gift of A. Fire), or obtained from the C. elegans whole genome RNAi feeding library (J. Ahringer, MRC Geneservice, Cambridge, United Kingdom).

The RNAi feeding strain was E. coli HT115 transformed with either empty pPD129.36 vector (controls) or with dsRNA-producing constructs. Plates were prepared as described in Kamath et al. (2001). Unless stated differently, wild-type N2 Bristol animals were plated as synchronized adults. To obtain synchronized worms of various stages, a large quantity of N2 gravid adults were collected, bleached, and grown to the required stage on HT115 that had been transformed with pPD129.36 (control).

#### GC analysis

A mixed population of well-fed worms were washed off the plates with water, rinsed 3–4 times, and, after aspirating away water, frozen at −80 °C. FA methyl esters and lipid extraction were performed as described in Miquel and Browse (1992). GC was performed on the HP6890N (Agilent, Palo Alto, California, United States) equipped with a DB-23 column (30 m × 250 μm × 0.25 μm) (Kniazeva et al. 2003). Each experiment was repeated at least five times. Average values and standard deviations were then calculated for each of the compounds in the experiments.

#### Staging worms to test for FA composition

After bleaching gravid adults, an aliquot of the eggs was set apart, and the rest was incubated overnight in M9 at room temperature. On the next day, an aliquot of L1 was frozen for GC analysis. The rest of L1 was plated on agar plates. Subsequently, L2, L3, L4, young adults, and adults along with hatched L1 were collected as the separate samples. Mixed populations of worms starved for 24–100 h were included in the experiment to monitor a possible effect of the starvation.

#### Phenotype rescue using FA supplements.

Ninety microliters of the 4 mM solution of FA (Sigma, St. Louis, Missouri, United States) in 1% NP40 was dropped on the side of the bacterial lawn that contained either *elo-5* dsRNA-producing plasmid or the control HT115 vector. Two synchronized young adults were plated and their progeny was scored 4 and 5 d later. Each experiment was performed in at least 30 replicates. For recovering *elo-5(RNAi)* worms from L1 arrest, wild-type adults were placed on the *elo-5(RNAi)* plates. Four days later, their progeny was removed and eggs of the next generation were left on the plates. Hatched L1 were kept for 2 or 4 d before transferring as agar chunks to new *elo-5(RNAi)* plates. FA supplements were added to spots next to the chunks. Ten plates were prepared for each FA supplement. Control plates contained no supplements. To verify that an addition of supplements did not affect RNA interference per se, we used *let-418(RNAi)* animals, which have a sterile phenotype, as a control. Neither C15 nor C17 mmBCFA added to *let-418(RNAi)* plates modified the expected phenotype.

#### Designing of GFP reporter constructs.

To prepare the GFP fusion constructs, genomic fragments were PCR amplified and cloned in frame into one of the GFP fusion vectors (gift of A. Fire). The locations of the genomic fragments and PCR primers used are listed below:

(1) *elo-5Prom*::*GFP,* starting at 3.894 kb genomic upstream of the first codon and ending 4 bp into the first exon; primers, F-BamHI-TTTAGGTCATTTTTTGAGTCGCCA and R-BamHI-TAGTCTGGAATTTTGAAATTGAACGG; vector, pPD95.69;

(2) *elo-6Prom*::*GFP,* a 4.764-kb fragment covering 3,104 bp upstream and 1,660 bp downstream of the predicted start codon and ending 14 bp into the third exon; primers, F-Sph1-GCCCTTGGAAACCATCTACGACGAATC and R-Sma1-TCCGAACAGAACGACATAAGAGATTTCC; vector, pPD95.77;

(3) *acs-1Prom*::*GFP,* a 3.142-kb genomic fragment containing 3,048 kb upstream of the first predicted ATG and ending 24 bp into the second predicted exon; primers, F-SphI-CATAATTACTATTGCGTCACATG and R-SphI-CTCTTCCAAACTGGCGATGTCGA; vector, pPD95.69;

(4) *pnk-1Prom*::*GFP,* a 1.14-kb fragment that includes 937 bp upstream of the first predicted codon of the C10G11.5 and 203 bp downstream, ending 24 bp into the second exon; primers, F-SphI-TCGTACGATCGGACCATAGGCTAA and R-SphI-CTGATCCTCTGTAGCAGCGGCCCT; vector, pPD95.69.

These constructs were injected into C. elegans at 10–50 ng/μl to form extrachromosomal arrays. In the case of *acs-1,* the extrachromosomal array had been integrated into the C. elegans genome.

#### Staining chemosensory ciliated neuron with DiI

Worms were soaked in a 5-μg/ml solution of DiI (Molecular Probes, Eugene, Oregon, United States) in M9 buffer for 1 h. They were then rinsed three times with M9 and visualized by fluorescence using the Texas Red filter.

#### Correlation analysis

The FA quantities obtained by GC were expressed as a percentage of the total. *t* test (two-tailed distribution) and correlation analysis were performed using the Microsoft Excel program.

#### Visualization and scoring of the GFP expression in promoter::GFP lines

Synchronized adults were placed on control (HT115 bacterial strain transformed with empty vector, pPD129.36) and RNAi (HT115 bacterial strain transformed with dsRNA construct) plates. Several worms of the next generation were picked from the control and RNAi plates and mounted on the same microscopic slide. GFP images were obtained with the fixed settings and exposure.

#### Microarray analysis

One young adult of the N2 Bristol strain was plated on each control and RNAi feeding plate. Control plates contained the E. coli HT115 strain transformed with empty pPD129.36 vector. Experimental RNAi plates contained E. coli HT115 transformed with corresponding dsRNA constructs. The growth conditions, RNA preparations, and data analyses are described in detail in Protocol S1. Expression data are presented in Dataset S1.

## Supporting Information

Dataset S1 Microarray Expression Data (1.8 MB TXT).

Figure S1 Expression of Collagen Genes as an Indicator of Developmental Differences in Mixed Populations of Worms Samples I, II, and III represent mixed populations of wild-type animals started simultaneously from one young adult. Each was harvested at three time points, when mostly adults represented the F1 generation and the embryos and larvae in different proportions represented the F2 generation (see Protocol S1). Sample III corresponds to the most diverse mixture of worms. Numbers of collagen genes that were differentially expressed between pairs of samples are shown above or bellow the arrow brackets. Sample I and Sample III, which originated from the most distal mixed populations, have the largest number of differentially expressed collagens. Sample I and an experimental sample corresponding to the *elo-5(RNAi)* phenotype have a lower number of the changed collagen genes, suggesting that populations on these experimental and control plates are similar.
(24 KB PPT).

Protocol S1 Microarray Data Analysis (37 KB DOC).

Table S1 Filtering Candidate Genes by Comparing Different Mutant and Wild-Type Samples (28 KB DOC).

## Abbreviations

ACC: acetyl-CoA carboxilase
BCAT: branched-chain aminotransferase
BCKAD: branched-chain α-keto-acid dehydrogenase
CoA: coenzyme A
dsRNA: double-stranded RNA
FA: fatty acid
FAS: fatty acid synthetase
G3PA: glycerol 3-phosphate acyltransferase
GC: gas chromatography
GFP: green fluorescent protein
mmBCFA: monomethyl branched-chain fatty acid
SI: saturation index
SREBP: sterol regulatory element binding protein
