# Supplementary figures and images for: Monomethyl Branched-Chain Fatty Acids Play an Essential Role in Caenorhabditis elegans Development
Source: PLoS Biol. 2004 Aug 31;2(9):e257. doi: 10.1371/journal.pbio.0020257 (PMC514883; doi:10.1371/journal.pbio.0020257)

## Slide 1
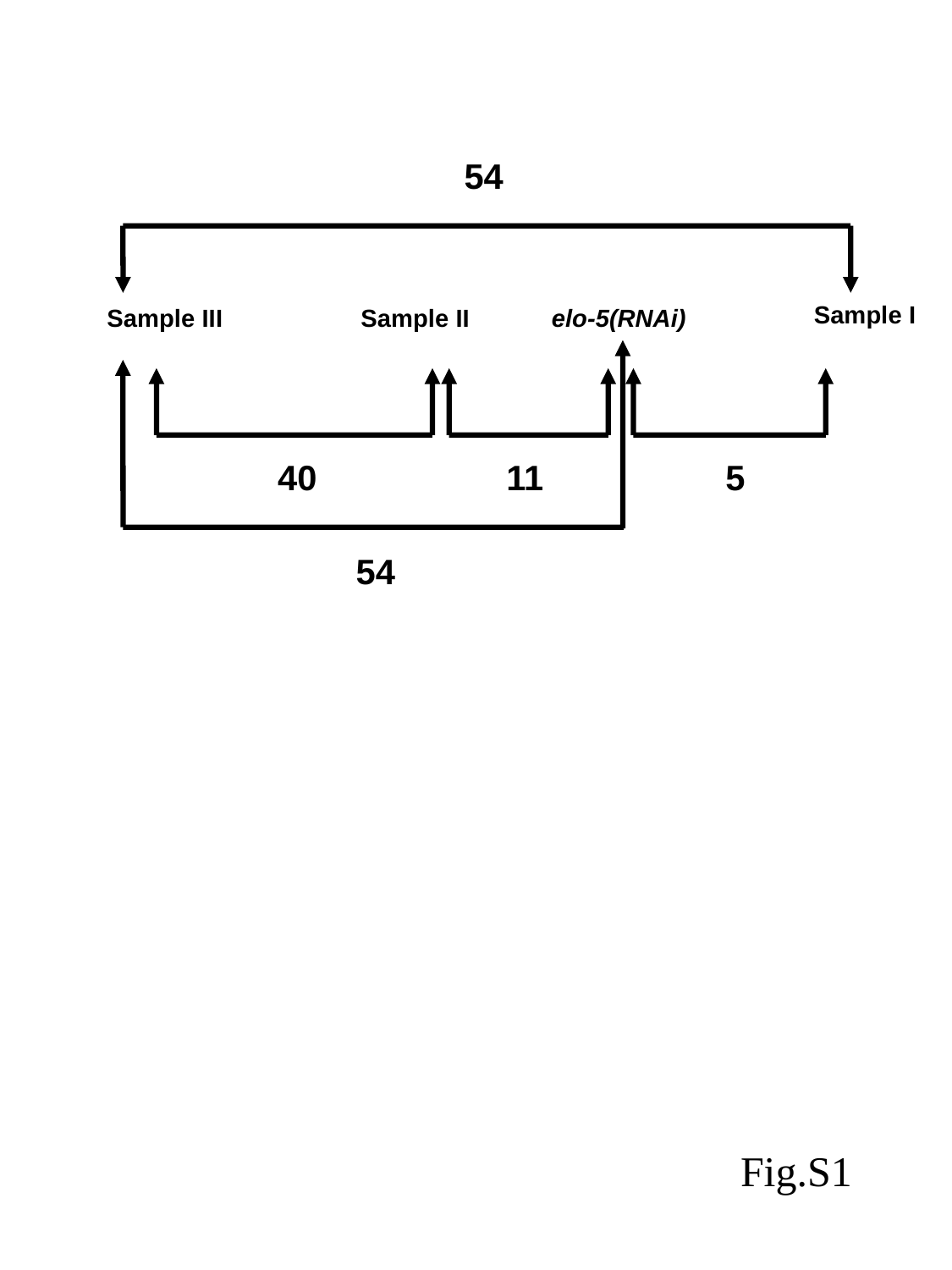

54
Sample I
Sample III
Sample II
elo-5(RNAi)
 40
 11
 5
 54
Fig.S1

Supplement: Figure S1 — Samples I, II, and III represent mixed populations of wild-type animals started simultaneously from one young adult. Each was harvested at three time points, when mostly adults represented the F1 generation and the embryos and larvae in different proportions represented the F2 generation (see Protocol S1). Sample III corresponds to the most diverse mixture of worms. Numbers of collagen genes that were differentially expressed between pairs of samples are shown above or bellow the arrow brackets. Sample I and Sample III, which originated from the most distal mixed populations, have the largest number of differentially expressed collagens. Sample I and an experimental sample corresponding to the elo-5(RNAi) phenotype have a lower number of the changed collagen genes, suggesting that populations on these experimental and control plates are similar. (24 KB PPT). [file pbio.0020257.sg001.ppt]
