# Supplementary material for: Monomethyl Branched-Chain Fatty Acids Play an Essential Role in Caenorhabditis elegans Development
Source: PLoS Biol. 2004 Aug 31;2(9):e257. doi: 10.1371/journal.pbio.0020257 (PMC514883; doi:10.1371/journal.pbio.0020257)
Supplement: Protocol S1 — (37 KB DOC). [file pbio.0020257.sd002.doc]

# Protocol S1.

**Microarray Data Analysis.**

Array design.

● We used GeneChip® *C. elegans* Genome Arrays (Cat.#900383 Affimetrix <http://www.affymetrix.com/products/arrays/specific/celegans.affx>) made with

in situ synthesized 25-mer oligonucleotides.

Samples used:

● Organism. *Caenorhabditis elegans*

● Strains. N2 Bristol and *elo-5(RNAi), spt-1(RNAi).*

● Sex. Hermaphrodites

● Age. Mixed.

● Organism parts. Whole animals.

● Quality control. Two replicate samples were obtained for each type of conditions (control and RNAi feeding). The samples were processed entirely independently in parallel experiments starting from plating the worms.

● One hybridization per a sample was used.

● 3’/5’ ratio for GAPDH and beta-actin were less then 3 in all hybridizations.

● Spike controls. BioB was called present and BioC, BioD, and CreX controls were present in increasing intensities in all hybridizations.

Experiment design:

● Aim. An identification of genes that change their expression level in response to a disruption of elongation of mono-methyl Branched-Chain Fatty Acid (mmBCFA) in *C. elegans*.

● Experimental conditions. Wild type N2 Bristol strain was compared with the *elo-5(RNAi)* strain. *elo-5* encodes the mmBCFA elongation enzyme essential for the mmBCFA biosynthesis.

● Growth conditions and preparation of control/reference samples. RNAi plates were prepared as described in (Kamath et al. 2001). The RNAi feeding strain was *E. coli* HT115 transformed with either the empty pPD129.36 vector (control, gift of A. Fire) or with the dsRNA-producing constructs. Worms were cultured at 20°C. One wild type young adult (P_0_) was placed on each plate. The population growth was monitored using a dissecting scope. Animals were harvested at three time points between 3^d^ and 4^th^ day after plating P_0_. To generate Sample I, worms were washed off the plates when the F1 population consisted of mostly adults and the F2 generation consisted of mostly L1, some L2 larvae and eggs. For Sample II, worms were washed off the plates several hours later when the F2 generation was enriched with L2. For Sample III, worms were maintained on plates until the F2 generation was represented by a mixture of L2, L3, and L4 larvae as well as some young adults.

● Growth conditions and Preparation of RNAi-treated samples. One wild type young adult was placed on each experimental RNAi plate. The *elo-5(RNAi)* and *spt-1(RNAi)* worms were harvested on 4^th^ day after plating P_0_ when the F1 generation consisted of mostly adults and the F2 generation consisted of mostly L1 larvae (or mostly L1 and L2 in the case of *spt-1(RNAi)*).

Total RNA isolation. Worms were collected in 15 ml conical tubes and rinsed 5 times in dH_2_0 followed by a treatment with the TRIAZOL reagent according to the manufacturer’s protocol.

Hybridization and data processing.

● RNA probes preparation were done according to the Affymetrix GeneChip®

Protocol in the University of Michigan Microarray Facility.

● The *C. elegans* GeneChip® (Affymetrix) hybridization were done according to the Affymetrix protocols on the company’s equipment in the University of

Michigan Microarray Facility.

Measurement data and specifications.

● Scanning hardware and software. Affymetrix GeneChip® Operating Software (GCOS) Version 1.0 was used for the control of GeneChip Fluidics Stations and Scanners, for data acquisition, sample management, for experimental information, and for gene expression data analysis.

Statistical algorithms.

● Microarray Suit v.5 (Affymetrix) software was used for single array analyses. It utilizes the One-sided Wilcoxon’s Signed Rank test as a statistical method to generate the Detection p-values and One-Step Turkey’s Biweight Estimate to calculate signals. We performed the global scaling (all probe sets) to the target intensity (TGT) of 100, suggested by Affimetrix® protocol, and filtered out signals that were less than 70 because it was the average signal obtained with the BioB probe defining the minimal sensitivity of the assay (Affimetrix® protocol) in some hybridizations. The signals with Detection p-value > 0.05 were also filtered out.

● The Data Mining Tool (DMT) software (Affymetrix) was used for the comparison analysis (experiment vs. baseline arrays). Unpaired T-test without corrections was utilized to estimate significance of the difference between two means, where mean is an average signal between replicates for each of controls and experiments. Change p-value >0.05 was chosen as a cut-off. Fold Change was calculated and the transcripts that have their expression level changed >1.57 fold were considered. This arbitrary cut off was relatively low yet potentially detectable in future conformation tests.

● Filtered data sets representing comparisons between two conditions were saved as Excel files. For further data manipulations we used MatLab program and custom-made scripts (A. Kniazev, personal communication).

Microarray data manipulation and analysis.

To simplify the task of finding genes differentially expressed in response to the *elo-5 RNAi*-treatment, but not in response to the stage-regulated differences, we identified the latter in our control samples. We compared control samples and found 1609 genes differentially expressed between the most distant Sample I and Sample III and 287 genes differentially expressed between Sample I and Sample II. The genes differentially expressed between stages were removed from the list of candidate genes (we realize that by doing this filtration we may loose some of potential candidate genes, but it is not a major concern for this first pilot analysis). Not surprisingly, the most populated group of the genes with changed expression is a family of collagens known to be heterochronic genes in *C. elegans* (Liu et al. 1995; Rougvie and Ambros 1995).

We used a number of differentially expressed collagens for a measurement of similarity between the samples; the smaller the number, the fewer developmental differences observed between the compared samples (Fig. S1). Using this “collagen-number” method, we chose Sample I as a reference (or baseline) control for the *elo-5(RNAi)* and *spt-1(RNAi)* experiments.

We then identified the genes that had changed the expression in both *elo-5(RNAi)* and *spt-1(RNAi)* samples when compared to the control (Sample I), after which we subtracted these from the list of the candidates (Table S1). This step excluded a number of genes that may have non-specific changes in their expression, due to a "general" sickness, for example.

Two hundred and nine genes ended up on the list of candidates that presumably changed their expression level in response to the RNAi-mediated suppression of *elo-5* (Table S1). We re-annotated the genes using the updates from WormBase (www.wormbase.org), NCBI (www.ncbi.nlm.nih.gov/BLAST, and [www.ncbi.nlm.nih.gov/Entrez](http://www.ncbi.nlm.nih.gov/Entrez)). After the analysis, 41 genes still remained unclassified.

For further analysis, we first selected 25 genes encoding proteins related to transcription regulation, lipid metabolic enzymes, intestinal and membrane proteins, and genes that were reported to have RNAi phenotypes similar to that of *elo-5(gk208)*.

In addition, we used the original data (genes differentially expressed in *elo-5(RNAi)* as compared to control Sample I minus developmentally regulated genes) as a reference in order to look for other potential genes of interest. In particular, we checked the expression of *lpd-1* and found that it is increased in *elo-5(RNAi)*. This gene was included in the short list of candidate genes.

Each of the candidates from the short list was functionally tested for its relationship with mmBCFA metabolism by RNAi followed by GC analysis of the FA composition.
