# Supplementary material for: Monomethyl Branched-Chain Fatty Acids Play an Essential Role in Caenorhabditis elegans Development
Source: PLoS Biol. 2004 Aug 31;2(9):e257. doi: 10.1371/journal.pbio.0020257 (PMC514883; doi:10.1371/journal.pbio.0020257)
Supplement: Table S1 — (28 KB DOC). [file pbio.0020257.st001.doc]

Table S1. Filtering candidate genes by comparing different mutant and wild-type samples.

420

420

Among the remaining genes, the number of genes differentially expressed in both RNAi samples

The number of genes differentially expressed in either RNAi sample

After excluding genes that expressed differentially between control

Sample I and Sample II (287) **^1^**

After excluding genes that expressed differentially between control Sample I and Sample III (1609)^1^

Total differentially expressed genes

(Signal>70, P-detection<0.05, P-change <0.05, Fold Change>1.57)

1317

209

1737

629

1805

669

2702

1312

*spt-1(RNAi)*

vs. wild type

(Sample I)

*elo-5(RNAi)*

vs. wild type (Sample I)

**^1^**Sample I, II and III represent populations of wild type worms started from synchronized young adults and harvested in three time points (see Supplemental Methods and Fig. S1). Sample I and Sample III originated from the most distant populations, with total 1609 genes differentially expressed between them. Sample I and Sample II are closer, with only 287 genes differentially expressed between them.
